# Nephrogenic adenoma of the bladder: a single institution experience assessing clinical factors

**DOI:** 10.1590/S1677-5538.IBJU.2017.0155

**Published:** 2018

**Authors:** Yooni Yi, Angela Wu, Anne P. Cameron

**Affiliations:** 1 Deparment of Urology, University of Michigan, Michigan, USA; 2 Deparment of Pathology, University of Michigan, Michigan, USA

**Keywords:** Urinary Bladder Neoplasms, Adenoma, Urologic Diseases

## Abstract

**Introduction:**

Nephrogenic adenoma (NA) was first described by Davis in 1949 as a “hamartoma” of the bladder. There are many proposed predisposing factors for NA including chronic inflammation, renal transplantation, and bladder cancer. We examined our experience with NA to determine predisposing factors and determine if there was any increased risk for development of subsequent malignancy.

**Materials and Methods:**

All patients with a pathologic diagnosis of bladder NA from 2001-2013 were included. Patient history, clinical factors including possible predisposing factors for NA, and follow-up were reviewed.

**Results:**

Among 60 patients, 68% were males with an average age of 61, an average BMI of 28.7, and 60% had a smoking history. In evaluating pro-inflammatory factors, 26.7% underwent either Bacillus Calmette-Guerin or mitomycin C, 30% had recurrent urinary tract infections, and 25% had a history of catheterization. Recurrence of NA after initial resection occurred only in 14.7% of patients who underwent follow-up cystoscopy. A history of concurrent bladder cancer was seen in 41.7% of patients, but there were no cases of *de novo* bladder cancer diagnosed after NA.

**Conclusion:**

To the best of our knowledge, this is the largest series of patients with NA of the bladder. NA occurs in a heterogeneous population of patients, but most often with underlying inflammation. NA occurred concurrent with bladder cancer; however there were no cases of *de novo* bladder cancer after NA, reassuring that NA is likely a benign reactive condition.

## INTRODUCTION

Nephrogenic adenoma (also called nephrogenic metaplasia) was first described in 1949 as a hamartoma of the bladder – a “tumor-like nodule of superfluous tissue retaining the basic structure of the tissue in which it was situated and differentiated from it by hyperplasia” (1). In 1950, the term ‘nephrogenic adenoma’ (NA) was coined in a case series of patients describing similar pathologic findings in the bladder (2). Small case series have reported NA occurring concurrently with bladder cancer (3, 4–7). Though TCC may be concurrent with the findings of nephrogenic adenoma, malignant transformation of nephrogenic adenoma has only been demonstrated in a few small case series (8–10). The predisposing factors for this lesion are poorly understood, though many studies will report prior trauma or inflammation to play a role in the development of NA such as recurrent urinary tract infections, chronic catheterization or administration of intravesical therapy (3–5, 7, 8, 10–15). Given its rarity, there are very few large series to assess these concerns and no clear guidance on its management and follow-up (16).

We examined our institution's experience with patients diagnosed with nephrogenic adenoma of the bladder to determine any predisposing factors for nephrogenic adenoma and also to de-termine if any patients subsequently developed a *de novo* bladder cancer.

## MATERIALS AND METHODS

This study was approved by our Institutional Review Board. Utilizing our institution's pathology database, we compiled all patients diagnosed with NA of the bladder from 2001-2013. Only patients with an initial, *de novo* diagnosis were included. NA of the upper tract or other locations other than the bladder was not included in this retrospective review. The pathology slides and compilation of pathology reports were completed by the genitourinary pathologists at our institution.

Possible predisposing factors as well as other clinical history were reviewed. These included prior catheterizations, urologic history, recurrent infections, history of transplant, and concomitant bladder cancer. Concurrent bladder cancer diagnosis was defined as a patient with nephrogenic adenoma and bladder cancer in the same specimen. We also tracked any patients with a *de novo* diagnosis of bladder cancer subsequent to their diagnosis of NA. Recurrence of NA was defined as a patient completely treated for NA with resection that subsequently developed a new lesion.

## RESULTS

Sixty patients with NA of the bladder were identified. The average age at diagnosis was 61 (range 8-91). There was only one pediatric patient. The majority of the population was male (68.3%) and had a smoking history (58.3%). There were only 3 (5%) patients with a history of renal transplant and 21 (35%) patients with a history of chronic kidney disease. All but two patients had prior urologic history including prostate cancer, interstitial cystitis, bladder cancer and neurogenic bladder (Table-1). Fifteen (25%) patients were found to have been intermittently catheterized or have a current indwelling Foley catheter and 18 (30%) patients had a prior history of recurrent urinarytract infections defined as greater than or equal to three infections in a year. The most common presenting symptoms were lower urinary tract symptoms (35%) and hematuria (28.3%) (Table-2).

**Table 1:** Patient Characteristics.

Gender		
	Male	68% (n=41)
	Female	32% (n=19)
Average BMI		28.7
Average age at diagnosis		61 years (range 8-91)
Smoking history		58% (n=35)
History of Bladder Cancer		41.7% (n=25)
**Trauma/Irritative Risk Factors**		
	History of Intravesical Therapy	26.7% (n=16)
	History of CIC/Catheter	26.7% (n=16)
	History of recurrent UTI	30% (n=18)

**BMI** = body mass index; **CIC** = clean intermittent catheterization; **UTI** = urinary tract infection

**Table 2:** Presenting Symptoms Prior to Cystoscopy.

Symptoms	Number of Patients
LUTS	21 (35%)
Hematuria	15 (25%)
Asymptomatic	9 (15%)
Incontinence	8 (13%)
Dysuria	4 (7%)
Recurrent UTI	4 (7%)
Urinary Retention	4 (7%)
Flank Pain	2 (3%)
Stone	1 (2%)
Other	1 (2%)

**LUTS** = lower urinary tract symptoms; **UTI** = urinary tract infection

The surgical specimens on which NA were diagnosed included 14 transurethral resections (TURs), 34 biopsies, and 10 cystectomies for concurrent advanced bladder. Previous bladder cancer history was present in 25 (41.7%) patients and 16 (26.7%) had undergone Bacillus Calmette-Guerin or mitomycin C instillations. Ten of the patients had NA diagnosed concurrently with their bladder cancer. Ten of the patients had NA diagnosed only on the cystectomy specimen with no prior NA diagnosis. Two other patients had cystectomies for invasive bladder cancer following their diagnosis of NA and known concurrent bladder cancer.

Of the 50 patients who had not undergone cystectomy, thirty-four patients had a follow-up cystoscopy. Recurrence was noted in 5 (14.7%) patients. Three of the patients had diffuse involvement of the bladder with NA at presentation and were likely incompletely resected while two of them had only singular small areas of NA. All were managed with biopsy and/or resection along with fulguration. In the setting of diffuse involvement of the bladder (more than 25% of the bladder surface involved) these often required multiple fulgurations for relief of symptoms and clearance of the lesions. None of these patients with recurrence had a history of bladder cancer or concurrent bladder cancer. Overall, despite the high proportion of patients with bladder cancer in this series, no patients were diagnosed with a bladder cancer after NA at an average follow-up of 10.1 months.

## DISCUSSION

**Figure 1:**
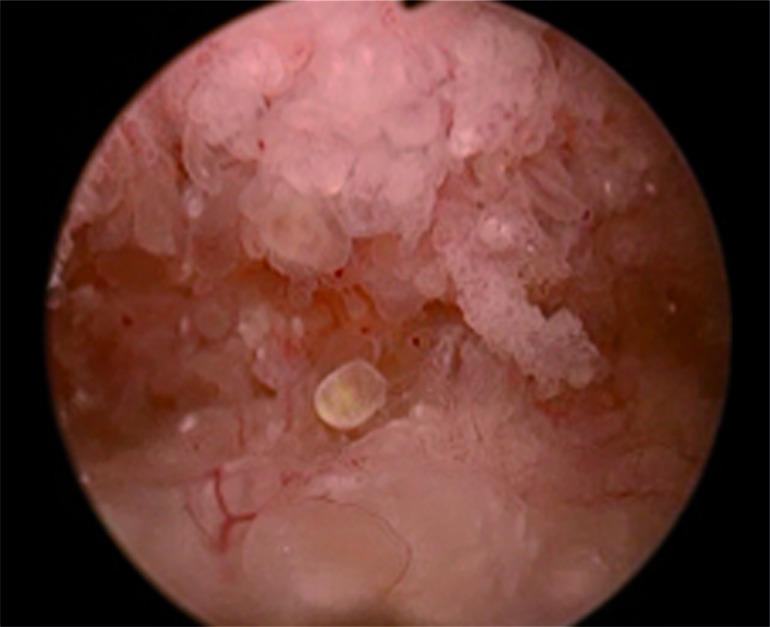
Cystoscopic findings of nephrogenic adenoma mimicking other urology pathology.

**Figure 2:**
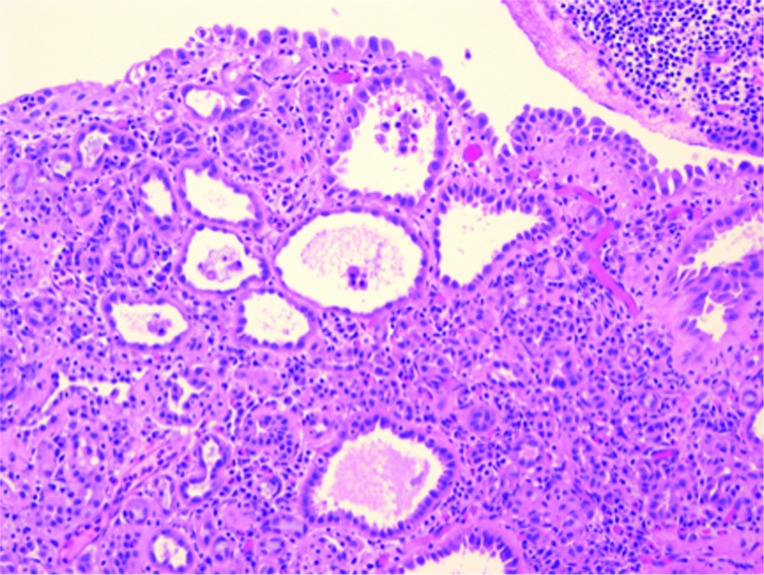
The papillary cores are lined by a single layer of cuboidal epithelium. They contain acing structures and dilated tubules.

NA is still considered a rare finding; however, reports of its occurrence have increased since its initial description in 1949 (Table-3). Direct cystoscopic visual findings of these lesions (Figure-1) are varied across multiple studies but often can mimic other urologic conditions such as urothelial carcinoma or chronic cystitis (16). There too exists variation in subtypes of the NA pathologic findings. Figure-2 shows an example of a pathology slide at 200 magnification showing papillary cores that are lined by a single layer of cuboidal epithelium which contain acinar structures and dilated tubules. To our knowledge, this is the largest clinical cohort of NA of the bladder published. This study found that the most common presentation of NA is lower urinary tract symptoms and hematuria, which according to AUA Guidelines would lead to an evaluation with a cystoscopy (17). Other presentations included retention, dysuria, recurrent UTIs and some were asymptomatic. The exact etiology of nephrogenic adenoma has not been identified, but there are reports of patients with renal transplantation being at risk for developing nephrogenic adenoma (3, 6). Mazal et al. looked at NA in patients with kidney transplantation and found that the cells originated from the donor kidney tubular cells and not from the urothelial mucosa. It is likely that seeding from the kidney of these renal-like cells is the same etiology in non-transplant patients (8).

**Table 3:** Summary of publications reviewing patients with nephrogenic adenoma of the bladder.

Authors	Publication Year	Study Population	Association with Bladder Cancer	Presenting Symptoms	Risk Factors	Recurrence	Conclusion
Kaswick et al. (13)	1976	2	No	Hematuria Incontinence	History of prior urosurgery History of UTI	1 recurrence	-treatment with TUR, antibiotics -monitor for recurrence
Molland et al. (15)	1976	3 patients	No	Unknown	Unknown	Unknown	Malignant transformation to adenocarcinoma in one patient
Ford et al. (4)	1985	70 (35 within bladder)	7 patients with TCC	Incidental findings	History of prior urosurgery	15 patients	No malignancy in 12 year follow up
Oliva et al. (5)	1995	80 (42 within bladder)	No	N/A	Prior urosurgery nephrolithiasis infection	N/A	NA with bland cytologic features
Peeker et al. (6)	1997	31 (24 within bladder)	7 patients with UCC	Hematuria Urinary frequency Bladder Pain	History of renal transplant (1) History of prior urosurgery History of UTI	7 patients	-Trauma to urothelial mucosa may lead to NA - TUR as method of management
Tse et al. (18)	1997	22	6 patients with TCC	Hematuria Urinary frequency Incidental	History of renal transplant (7) Recurrent UTI History of prior urosurgery	6 patients	With association to TCC, NA should be taken seriously and followed up
Porcaro et al. (12)	2001	8	3 patients with TCC	Hematuria Irritative voiding symptoms	History of prior urosurgery	5 patients	-Features of NA nonspecific but TUR will diagnose & treat -high recurrence rate requiring follow up
Chen et al. (11)	2006	8	No	Hematuria Urinary frequency	History of urosurgery History of catheterization History of UTI	3 patients (median relapse 7 months)	-NA is a benign lesion -recurrence is high
Hungerhuber et al. (19)	2008	1	Progression to adenocarcinoma	Hematuria	History of urosurgery	Yes	NA may have malignant potential and warrants follow up
Dhaliwal et al. (10)	2012	1	Progression to Clear cell adenocarcinoma	Hematuria Proteinuria	History of urosurgery	No	Malignant transformation can occur with NA
Kuzaka et al. (3)	2014	3	1 patient with TCC	Hematuria	History of recurrent UTI History of prior urosurgery	2 patients (5 and 9 months)	-NA is a benign lesion -Recurrence is high
Gordetsky et al. (7)	2016	31 (26 within bladder)	12 patients with UCC	Hematuria Urinary incontinence Hydronephrosis Asymptomatic	History of renal transplant (1) History of DM (8 patients)	1 patient	-NA is a benign lesion

**TCC** = transitional cell carcinoma; **N/A** = not applicable; **NA** = nephrogenic adenoma; **UCC** = urothelial cell carcinoma; **TUR** = transurethral resection; **DM** = diabetes mellitus

Similar to the majority of other studies, we found a male predominance (2, 5, 7, 11, 12). Though this study focused on NA of the bladder, other studies have assessed other locations but all showed bladder as the most common location (4, 5, 11, 12). Similar to prior studies most of our patients had well-defined urologic issues prior to the diagnosis, including recurrent urinary tract infections, benign prostatic hyperplasia, interstitial cystitis or urothelial cell carcinoma (3–5, 7, 8, 13–15).

The vast majority of studies have supported that NA is a benign lesion (3–5, 7, 11); NA is not associated with deep invasion, mitotic activity, neoplastic-type atypia, or de-differentiation. NA generally grows slowly, and there has been no convincing evidence of any malignant transformation of NA (1, 2, 4, 11, 12).

Though multiple studies demonstrate the benign pathologic findings along with clinical absence of progression to malignancy (2, 4, 5, 11, 12, 18), a single study reported a case of nephrogenic adenoma that ultimately developed into adenocarcinoma 2 years later with later findings of metastatic adenocarcinoma. They refer to studies in which the association between nephrogenic metaplasia and clear cell adenocarcinoma was seen in the Pax 8 staining seen in both but not with urothelium, prostatic adenocarcinoma or urothelial carcinoma. This case study, however, may instead represent what has been described as a clear cell adenocarcinoma with diffuse areas mimicking NA (8). Other studies have also reported the possible relation between nephrogenic adenoma and mesonephric adenocarcinoma warranting aggressive follow-up and management, but these studies are rather old (9, 19). Overall, the vast majority of the recent literature, including our study, supports that NA is a benign reactive lesion with no associated increased risk of the development of bladder cancer.

There are no guidelines at present on the management and follow-up of nephrogenic adenoma. Recurrence rates have differed across studies. Our study showed recurrence in five patients and Gordetsky's recent study found one in 31 patients with recurrence. Other studies had varying numbers of recurrence, but recommendations were made to have long term follow-up as recurrences were common (5–8, 13, 14, 20). Given the symptomatic nature of this lesion and its tendency to recur, it would be prudent to follow patients with a cystoscopy and treat any of these recurrences. The exact timeline of cystoscopic follow-up is unknown, but based on the studies reviewed, patients were already on a surveillance schedule based on their history of bladder cancer and/or they presented with recurrent symptoms of hematuria or lower urinary tract symptoms which would prompt a re-evaluation. Follow-up noted in each of the studies varied largely from 2-24 months with reported average recurrence occurring within the first year (3, 13). We would recommend standard follow-up for bladder cancer patients. A possible plan for patients with nephrogenic adenoma alone may be a follow-up cystoscopy within 6-12 months from the initial diagnosis and then with symptom recurrence. Larger studies with longer follow-up will best answer this question.

Based on our review, this is the largest study of a cohort of 60 patients with an average 10.1 month follow up of patients with nephrogenic adenoma of the bladder. However, we do re-cognize there are limitations to the conclusions from this study. This is a retrospective chart re-view of patients identified by our pathology database. Our average follow-up was only 10.1 months, and only 68% of the population with intact bladders underwent follow-up cystoscopy. There is also likely a selection bias favoring more severe disease involvement as likely only symptomatic patients and/or patients with a history of bladder cancer would undergo cystoscopy and there are likely asymptomatic patients in the community who have small non-symptomatic lesions that we do not capture.

## CONCLUSIONS

In conclusion, patients with nephrogenic adenoma represent a heterogeneous population with varying presentations that cover the wide span of urologic subspecialties. NA has not been shown to lead to malignant progression in this study and in the majority of the literature. There is a predilection for patients that are male with priorurologic conditions; the most common presenting symptoms being lower urinary tract symptoms or hematuria. Recurrence does occur, but was noted to be relatively uncommon in this study. As for management, patients who are under surveillance for bladder cancer should continue the surveillance schedule with biopsy/resection of lesions that may appear suspicious as per usual regimen. Patients with a diagnosis of only nephrogenic adenoma should likely undergo repeat cystoscopy to confirm eradication of the lesions after resection, but given the limited data a follow-up regimen is difficult to establish.
